# Differential Expression of DNA Repair Genes in Prognostically-Favorable versus Unfavorable Uveal Melanoma

**DOI:** 10.3390/cancers11081104

**Published:** 2019-08-02

**Authors:** Mehmet Dogrusöz, Andrea Ruschel Trasel, Jinfeng Cao, Selҫuk Ҫolak, Sake I. van Pelt, Wilma G. M. Kroes, Amina F. A. S. Teunisse, Samar Alsafadi, Sjoerd G. van Duinen, Gregorius P. M. Luyten, Pieter A. van der Velden, Adriana Amaro, Ulrich Pfeffer, Aart G. Jochemsen, Martine J. Jager

**Affiliations:** 1 Department of Ophthalmology, Leiden University Medical Center, 2333 AZ Leiden, The Netherlands; 2 Department of Ophthalmology, Amsterdam University Medical Center, 1105 AZ Amsterdam, The Netherlands; 3 Universidade Federal do Rio Grande do Sul, 90040-060 Porto Alegre, Brazil; 4 Department of Ophthalmology, The Second Hospital of Jilin University, Changchun 130012, China; 5 Department of Molecular Cell Biology, Leiden University Medical Center, 2333 AZ Leiden, The Netherlands; 6 Center for Reproductive Medicine, Elisabeth-TweeSteden Hospital, 5022 GC Tilburg, The Netherlands; 7 Department of Clinical Genetics, Leiden University Medical Center, 2333 AZ Leiden, The Netherlands; 8 Department of Translational Research, PSL Research University, Institute Curie, 75248 Paris, France; 9 Department of Pathology, Leiden University Medical Center, 2333 AZ Leiden, The Netherlands; 10 Laboratory of Tumor Epigenetics, Department of Integrated Oncology Therapies, IRCCS Ospedale Policlinico San Martino, 16133 Genoa, Italy

**Keywords:** uveal melanoma, oncology, DNA repair, DNA-PK, PRKDC, BAP1, prognosis

## Abstract

Expression of DNA repair genes was studied in uveal melanoma (UM) in order to identify genes that may play a role in metastases formation. We searched for genes that are differentially expressed between tumors with a favorable and unfavorable prognosis. Gene-expression profiling was performed on 64 primary UM from the Leiden University Medical Center (LUMC), Leiden, The Netherlands. The expression of 121 genes encoding proteins involved in DNA repair pathways was analyzed: a total of 44 genes differed between disomy 3 and monosomy 3 tumors. Results were validated in a cohort from Genoa and Paris and the The Cancer Genome Atlas (TCGA) cohort. Expression of the *PRKDC*, *WDR48*, *XPC*, and *BAP1* genes was significantly associated with clinical outcome after validation. *PRKDC* was highly expressed in metastasizing UM (*p* < 0.001), whereas *WDR48*, *XPC*, and *BAP1* were lowly expressed (*p* < 0.001, *p* = 0.006, *p* = 0.003, respectively). Low expression of *WDR48* and *XPC* was related to a large tumor diameter (*p* = 0.01 and *p* = 0.004, respectively), and a mixed/epithelioid cell type (*p* = 0.007 and *p* = 0.03, respectively). We conclude that the expression of *WDR48*, *XPC*, and *BAP1* is significantly lower in UM with an unfavorable prognosis, while these tumors have a significantly higher expression of *PRKDC*. Pharmacological inhibition of DNA-PKcs resulted in decreased survival of UM cells. *PRKDC* may be involved in proliferation, invasion and metastasis of UM cells. Unraveling the role of DNA repair genes may enhance our understanding of UM biology and result in the identification of new therapeutic targets.

## 1. Introduction

Uveal melanoma (UM) is an ocular malignancy that arises from melanocytes residing in the uveal tract, which consists of the iris, ciliary body and choroid. It is the second most common type of melanoma and the most common primary intraocular malignancy in adults, affecting approximately 5.1 individuals per million per year; it is most frequent in Caucasians [1,2], as a fair skin and light eye color have been identified as host susceptibility factors [3,4].

In general, local tumor control is excellent, with large primary ocular melanoma being treated by enucleation, and small- to medium-sized tumors by application of a radioactive plaque, stereotactic irradiation or proton beam therapy [5,6,7,8,9,10]. Despite excellent regional tumor control, UM is still often lethal: up to 50% of patients will develop metastatic disease, for which no effective treatment exists [11]. The liver is involved in approximately 90% of cases with metastasized disease [12]. Metastatic disease may develop at any time from the initial diagnosis of the primary tumor to several years after diagnosis [13].

Several pathological characteristics of the primary tumor are known to be associated with an infaust prognosis. These include a large size, ciliary body involvement, epithelioid cell type, extrascleral invasion and the presence of extravascular matrix loops [14,15,16,17,18]. Furthermore, specific genetic features, such as monosomy 3, amplification of chromosome 8q, and loss of chromosome 1p, correlate with poor survival [19,20,21,22,23]. On the contrary, an additional copy of chromosome 6p is associated with a favorable prognosis [24,25]. Microarray gene expression analyses have resulted in the identification of two classes of UMs: class 1 tumors have low metastatic risk, while class 2 tumors are associated with a high rate of metastatic death [26,27,28].

Recently, mutations in specific genes such as *BAP1* (BRCA1-associated protein-1), *SF3B1* (splicing factor 3b subunit 1), and *EIF1AX* (eukaryotic translation initiation factor 1A, X-linked) have been reported to have prognostic value [29,30,31]. Aberrant DNA repair during the evolution of many malignancies and, accordingly, genomic instability is considered a hallmark of cancer cells [32]. Recent research in UM has focused on genetics, with the aim of unraveling UM biology and identifying specific aberrations that underlie the development of UM and may indicate potential targets of therapy [29,30,31,33]. The BAP1 protein, the loss of which correlates to a poor prognosis in UM [29,34,35], has been shown to promote DNA double-strand break repair [36]. Yet, the role of DNA repair in tumor development and progression remains poorly studied. Although counterintuitive, DNA-repair proteins in compensating pathways may be targets for cancer therapeutics [37,38]. Since tumor cells that have lost a repair pathway may (over)rely on them (principle of synthetic lethality), one may try to block DNA-repair proteins to decrease the ability of UM cells to repair DNA damage. This may subsequently help to sensitize tumors to traditional anti-cancer treatment by chemotherapy or radiotherapy [39].

However, it is not yet known whether and how the DNA-repair pathways are involved in the initiation and progression of UM. We, therefore, set out to analyze the expression of genes involved in DNA repair in UM and looked for genes that were associated with prognosis in UM.

To test our hypothesis that genes involved in DNA repair are differentially expressed between tumors with a favorable and unfavorable prognosis, we determined the expression of such genes in 64 UMs and made a comparison between tumors with and without loss of chromosome 3. Additionally, the relation with survival was evaluated for differentially expressed genes. Interesting associations were validated in two other sets of UM and a potential druggable target was explored further.

## 2. Results

### 2.1. Population Characteristics

Our cohort included 64 UM patients who had undergone primary enucleation at a median age of 61.6 years and of whom 33 (52%) were males (Table 1). The median largest basal diameter (LBD) was 13.0 mm and the median thickness 8 mm. Most tumors were either classified as American Joint Committee on Cancer (AJCC) tumor size T2 (39%) or T3 (48%). A mixed/epithelioid cell type was recorded in 66% of cases. Monosomy 3 was detected in 63% of the tumors. At last follow-up, 37 (58%) of patients had developed clinical metastases. We validated our data using two other independent cohorts: a set of 110 tumors from Genoa [40] and Paris [41], and the 80 UMs of The Cancer Genome Atlas (TCGA) project [42]. The characteristics of all cohorts are depicted in Table 1.

### 2.2. Gene Expression in Relation to Chromosome 3 Status

As loss of one copy of chromosome 3 is a very important prognostic marker in UM, we searched for DNA-repair-related genes that showed differential expression between tumors with and without loss of one chromosome 3.

**Figure 1:**
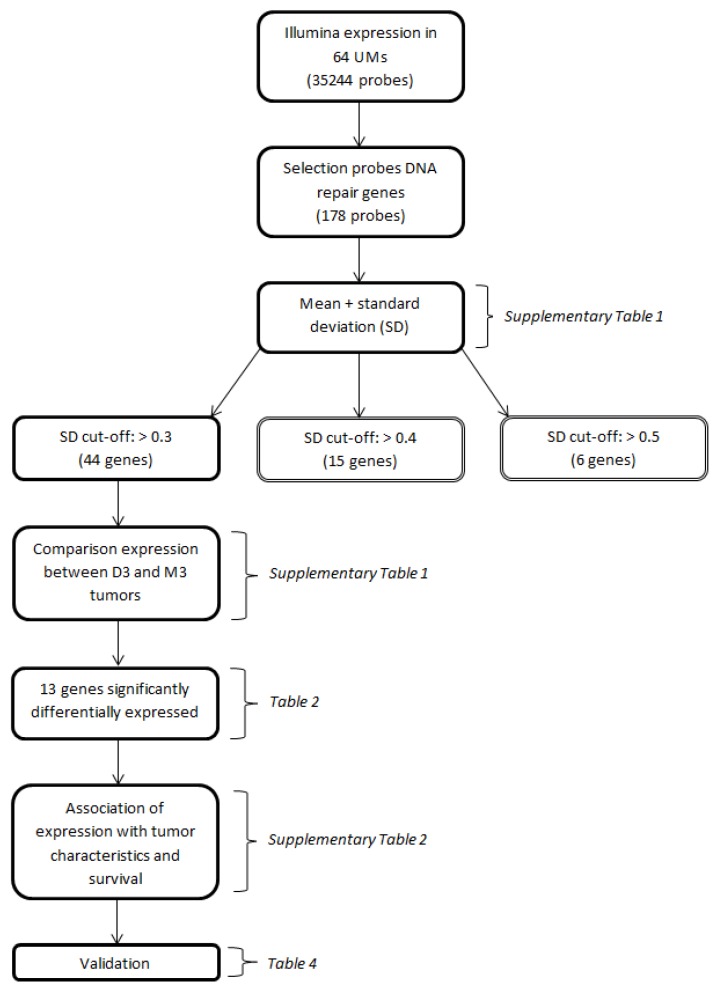
Flow-chart depicting the major conducted analyses. Parentheses indicate the tables in which the results of the respective analyses are presented.

We identified 121 genes encoding proteins involved in DNA repair mechanisms, based on a literature review on DNA repair, using the platforms Gene, OMIM, KEGG and PubMed. As our goal was to identify genes with a variable expression level, we determined the standard deviations of the expression levels of the DNA-repair-gene probes on the Illumina chip (*n* = 178) (Appendix Table A1). A selection of genes was made based on a cut-off value of the standard deviation of the expression (Figure 1).

The median expression of the 44 genes of interest was calculated and compared between disomy 3 and monosomy 3 tumors. Thirteen genes were significantly differentially expressed: three genes (*CENPX*, *DDB1*, *PRKDC*) were significantly higher in monosomy 3 tumors (Table 2A), while ten genes (*APEX1*, *BAP1*, *CETN2*, *GTF2H4*, *MLH1*, *RMI2*, *RPA1*, *SEM1*, *WDR48*, *XPC)* showed a significant down-modulation in tumors with monosomy 3 (Table 2B).

### 2.3. Gene Expression in Relation to Histological Data and Survival

The expression of the 13 genes that were differentially expressed between disomy 3 and monosomy 3 tumors was compared to histopathological data and survival (Appendix Table A2).

With regard to associations between gene expression and tumor diameter, we noticed an association between low expression of WDR48 and XPC and a large LBD (*p* = 0.01 and *p* = 0.004, respectively), while a high expression of CENPX correlated with a large LBD (*p* = 0.02). Although the difference in expression was small, *CENPX* showed a significantly higher expression in tumors with a mixed/epithelioid cell type (*p* = 0.04). On the contrary, the expression of the genes *WDR48* (*p* = 0.007) and *XPC* (*p* = 0.03) was significantly lower in cases with a mixed/epithelioid cell type. Regarding AJCC size categories, the expression of *CENPX* (*p* = 0.01) was significantly higher in tumors with higher AJCC categories, while the expression of the *RPA1* gene (*p* = 0.03) was significantly lower in cases with a higher AJCC category. The genes *CENPX* and *PRKDC* were highly expressed in tumors that gave rise to metastases (both *p* < 0.001). The genes *BAP1*, *CETN2*, *GTF2H4*, *MLH1*, *RMI2*, *SEM1*, *WDR48*, and *XPC*, instead, showed a lower expression in the metastasis group (Mann–Whitney U test). Considering survival, *CENPX* and *PRKDC* genes were associated with poor survival when highly expressed. A low expression of the genes *BAP1*, *GTF2H4*, *RMI2*, *SEM1*, *WDR48*, and *XPC* was instead associated with an unfavorable prognosis (log-rank test).

### 2.4. Chromosome Dose Effect and Expression Levels

As previously noticed for other genes, the loss or gain of chromosomal material might influence gene expression levels [43,44]. Therefore, we combined gene expression levels of all the 44 genes of interest with the SNP copy number value of the chromosome region harboring the gene (Table 3). We divided tumors into three groups: no aberration in the specified chromosome area, duplication in the specified region or deletion of the region of interest. The analysis was only reliable for genes located on chromosomes 3, 6 or 8, since SNP analyses of other chromosomes showed no aberrant copy number in most tumors.

As we expected, the four genes located on chromosome 3p (*BAP1*, *MLH1*, *WDR48*, *XPC*) showed an association between a decreased expression and presence of monosomy 3, while a trend towards decreased expression was noted for *MBD4* (chromosome 3q) (Table 3). The genes *FANCE* and *GTF2H4* (chromosome 6p) showed a significantly increased expression in tumors with a gain of 6p, while for *GTF2H5* (chromosome 6q), a significantly lower expression was found in tumors with loss of 6q. The expression of *POLB* (chromosome 8p) was significantly decreased in tumors with loss of 8p, while an increased expression of *NBN* and *PRKDC*, which are located on the long arm of chromosome 8, was related to a gain of genetic material in that chromosome region.

### 2.5. Validation

The 13 genes statistically differentially expressed were validated on the datasets of two other UMs: a set of 110 tumors from Genoa and Paris and another set of 80 UMs of The Cancer Genome Atlas (TCGA) project [42]. In each validation set, median expression levels for every gene were calculated to establish two groups of tumors for Kaplan–Meier analyses. The occurrence of metastases was the event of interest in the tumors from Genoa and Paris (taken together), while death due to UM metastases was the endpoint of analysis for the TCGA tumors. In Genoa and Paris sets, more than one *p*-value is presented for some genes, since several probes were available for these genes.

**Figure 2:**
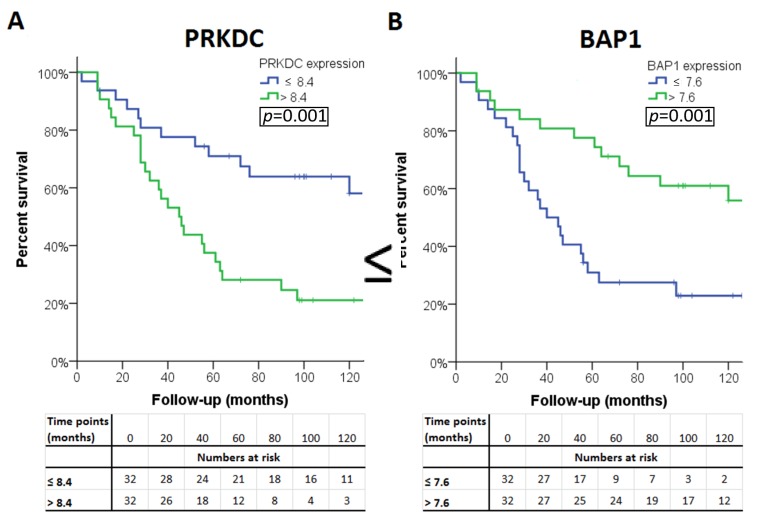

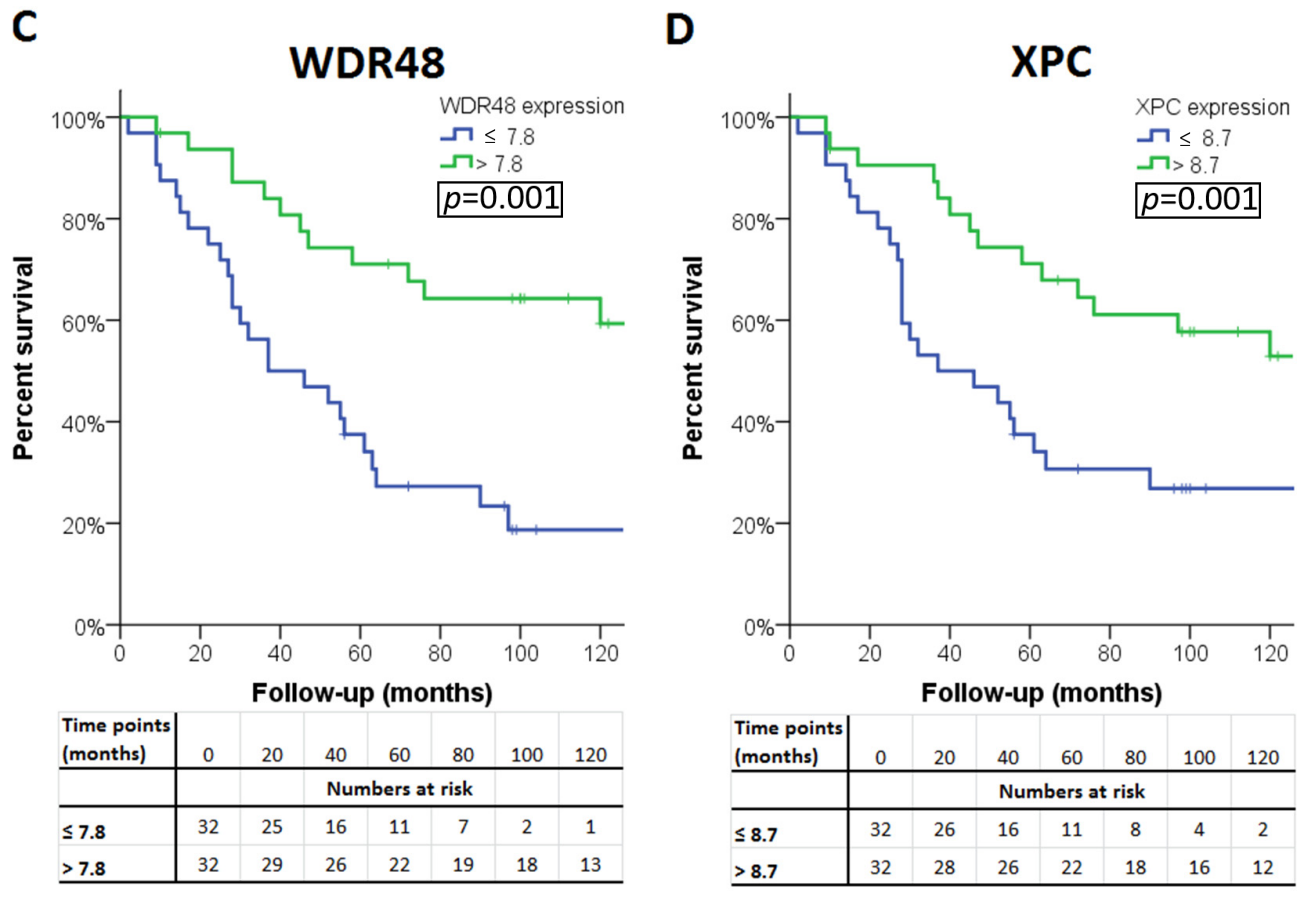
Kaplan–Meier survival curves of the four genes: (**A**) PAKDC, (**B**) BAP1 (**C**) WDR48 and (**D**) XPC significantly associated with clinical outcome in all three cohorts.

The association of the expression of a gene with survival was considered ‘validated’ if a significant association was observed in all three sets (LUMC set and the two validation sets). Four of the 13 genes were significantly associated with survival in all three cohorts. A high expression of *PRKDC* was associated with poor survival, as well as a low expression of *BAP1*, *WDR48*, and *XPC* (Table 4). Survival curves for these genes in patients from the LUMC cohort are shown in Figure 2. As cut-off value, we used the median expression of each gene.

### 2.6. PRKDC

Considering that a high expression of the *PRKDC* gene located on chromosome 8q is related to an unfavorable prognosis and the fact that gain of material of chromosome 8q predicts an adverse clinical outcome, we decided to perform further (experimental) analyses to study the biological significance of the *PRKDC* gene in UM. Our decision to focus on *PKRDC* was furthermore fueled by the finding that the DNA-PKcs protein encoded by *PRKDC* has been shown to modulate cell survival, proliferation, invasion and migration in other cancers [45,46].

**Figure 3:**
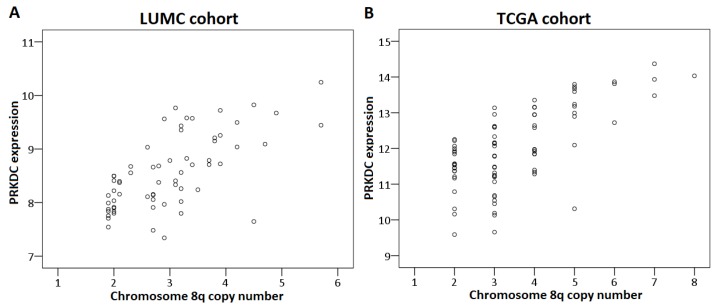
Correlation between *PRKDC* expression and chromosome 8q copy number in primary UM. The Spearman’s correlation test was performed. (**A**) LUMC cohort, (**B**) TCGA cohort.

First, we analyzed the relation between chromosome 8q copy number variation and *PRKDC* expression in the LUMC and the TCGA cohort. This analysis could not be performed for the Genoa and Paris cohort because the chromosome 8q status of these tumors was unknown. A higher chromosome 8q copy number was significantly correlated to a higher expression of *PRKDC* in the LUMC cohort (correlation coefficient: 0.67, *p* < 0.001) as well as the TCGA cohort (correlation coefficient: 0.61, *p* < 0.001) (Figure 3).

**Figure 4:**
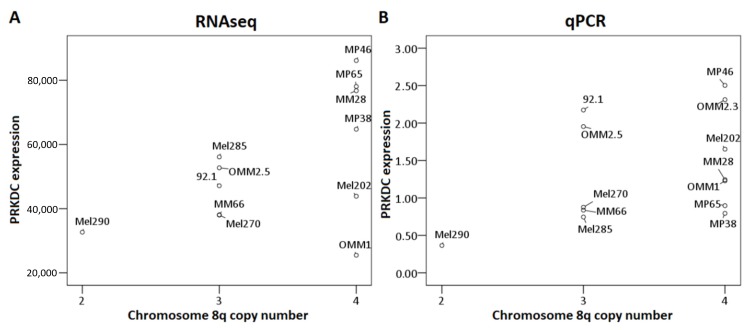
Association between PRKDC expression and chromosome 8q copy number in UM cell lines. The Kruskal–Wallis test was performed. (**A**): RNAseq, (**B**): qPCR.

We also analyzed the association between 8q copy number and *PRKDC* expression, determined by RNAseq in 12 UM cell lines, and by qPCR in 13 UM cell lines (Figure 4). Although the association was not significant (RNAseq: *p* = 0.23, qPCR: *p* = 0.2 [Kruskal–Wallis test]), we observed a trend towards higher expression of *PRKDC* in cell lines with more copies of 8q, which was in agreement with our findings in primary tumors (Figure 3). However, this association was less evident than in primary tumors, due to the lower number of cases and the lack of cell lines with two copies of chromosome 8q or more than four copies of 8q. The correlation was most pronounced in the RNAseq analysis (Figure 4A) and less clear in the qPCR analysis (Figure 4B), where the correlation was slightly distorted by cell lines 92.1 and OMM2.5, which have three copies of chromosome 8q but show a *PRKDC* expression that is comparable to cell lines with four copies. However, there was a subpopulation of cells having four copies of chromosome 8q in cell line 92.1, indicating mosaicism.

**Figure 5:**
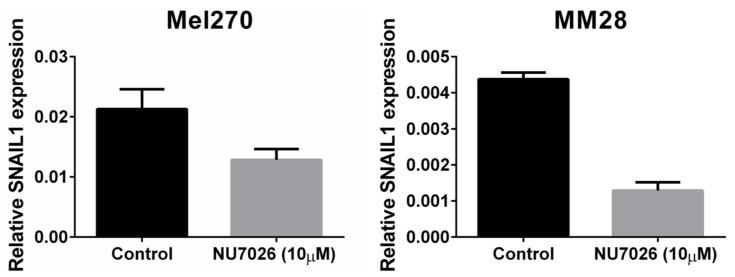
The effect of DNA-PKcs inhibition on the mRNA expression of *SNAIL1* in cell lines Mel270 and MM28. Cells were treated with 10 µM of the DNA-PKcs inhibitor NU7026 for 5 days.

**Figure 6:**
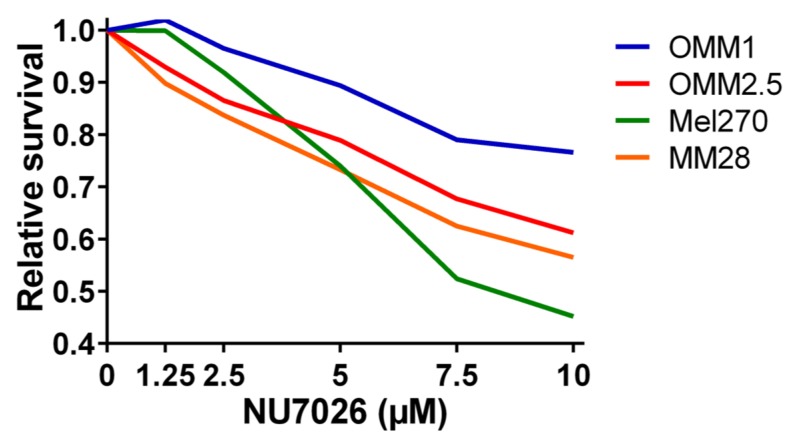
The relative survival in UM cell lines OMM1, OMM2.5, Mel270, and MM28 upon treatment with increasing doses of the DNA-PKcs inhibitor NU7026 for 5 days.

To test our hypothesis that *PRKDC* is a possible driver of metastasis in UM, we wondered in which ways *PRKDC* could be involved in invasion and migration of UM cells. A study in prostate cancer showed that transcriptional regulation by the DNA-PKcs protein encoded by the *PRKDC* gene promotes invasion, migration and metastasis [45]. As the expression of *ZEB1*, *TWIST1* and *SNAIL1* have been proposed to play a role in invasion of UM cells, [47] we evaluated whether inhibition of DNA-PKcs with NU7026 would influence the expression of these genes. NU7026 is an inhibitor of DNA-dependent protein kinase (DNA-PK), an enzyme involved in the non-homologous end-joining (NHEJ) DNA-repair pathway [48]. NU7026 sensitizes cells to radiation and has potential for use in anticancer therapies [49,50]. The expression of *ZEB1*, *TWIST1* and *SNAIL1* was evaluated in a primary UM cell line (Mel270) and in a metastatic UM cell line (MM28) before and after treating the cells with 10μM NU7026 for 5 days. The basal expression level of these genes was low in both cell lines. Inhibition of DNA-PKcs by NU7026 led to a downregulation of *SNAIL1* in Mel270 as well as MM28 cells (Figure 5). *ZEB1* and *TWIST1* expression were not affected. To analyze the effect of DNA-PKcs inhibition on cell proliferation, we treated four cell lines (OMM1, OMM2.5, Mel270, MM28) with increasing doses of NU7026 up to 10 µM for a period of 5 days (Figure 6). The proliferaton of all cell lines was affected by the DNA-PKcs inhibitor. The strongest growth inhibitory effect was noted in cell lines Mel270 and MM28, showing a 55% and 43% inhibition, respectively.

## 3. Discussion

Biological cellular responses following DNA damage include DNA damage repair, damage tolerance, cell-cycle checkpoint control and apoptosis. These mechanisms are tightly regulated and which pathway becomes activated depends on the type and severity of the DNA damage. In case of severe damage, the complex signaling pathways may eventually lead to cell cycle arrest (providing the cell more time for repair and tolerance mechanisms) or to apoptosis [51,52]. The recognition of expression patterns of the genes involved in DNA repair in UM is the first step in understanding the way these genes might play a role in UM development and may help in identifying new targets for therapy. We evaluated the expression of DNA-repair-related genes in the Leiden cohort of 64 UMs and aimed to identify genes with a variable expression between prognostically favorable and prognostically unfavorable UM. After validation in two other independent cohorts, we identified four genes which were associated with the degree of malignancy in UM: three genes (*BAP1*, *WDR48*, and *XPC1*) showed an association between a low expression and poor survival, while *PRKDC* was highly expressed in cases with an unfavorable prognosis. The genes *BAP1*, *WDR48*, and *XPC1* are all located on chromosome 3p and showed a significantly lower expression in monosomy 3 tumors. A lower expression of the *MLH1* gene, which is also located on chromosome 3p, was significantly related to prognosis in one cohort and showed a near-significant effect in the other cohorts. Since these four genes play a role in DNA repair, we can expect that impaired DNA repair is one of the results of the loss of a copy of chromosome 3. Sustained DNA damage as a result of deficient DNA repair mechanisms may lead to the accumulation of chromosomal abnormalities and gene mutations, which may promote cell growth and proliferation. Chromosome 3 loss does not occur in a single step since small tumors with partial monosomy have been observed [53], but apparently, loss of the entire chromosome confers a selective advantage that might be mediated by the DNA-repair genes identified here.

*BAP1* (BRCA1-associated protein 1) is a gene located on chromosome 3p21.1. The *BAP1* gene encodes a nuclear ubiquitin carboxy-terminal hydrolase, which is a deubiquitinating enzyme [54]. It has been described to be a tumor suppressor gene in the BRCA-1 control pathway. The BAP1 protein contains binding domains for BRCA1 and BARD1, enzymes that form a heterodimeric complex that functions as a tumor suppressor [55]. Loss of BAP1 has been shown to be related to a poor clinical outcome in UM [29]. Similarly, a lower gene expression of *BAP1* in our study corresponded to a poor survival.

Ubiquination and deubiquination regulate essential biological processes such as DNA replication and DNA repair [42,55]. In accordance, BAP1 has been shown to play a role in the repair of DNA double-strand breaks by homologous recombination [36,56]. It has been suggested that the DNA-repair function of BAP1 may be the molecular basis for its tumor suppressor role in UM [36].

Another DNA-repair-related gene involved in deubiquitination, which in our study showed a low expression in metastasizing uveal melanoma, is *WDR48.* It is also known as *UAF1* and is located in close proximity (on 3p22.2) to *BAP1*. UAF1 and USP1, a deubiquitinating enzyme, form the UAF1/USP1 complex, which regulates the Fanconi Anemia DNA-repair pathway [57]. UAF1 activates USP1, and USP1 regulates the Fanconi Anemia repair pathway by deubiquitinating FANCD2, one of the most important players in this pathway. Fanconi Anemia is an inherited genomic instability disorder that led to the discovery of a novel DNA-repair pathway. The Fanconi Anemia repair pathway plays a role in the repair of DNA cross-links and can be activated after various types of DNA damage, such as ionizing radiation and ultraviolet light [58,59]. Accurate deubiquitination of the FANCD2 protein by the USP1/UAF1 complex is essential for an intact Fanconi Anemia pathway and proper DNA damage repair [60,61]. Because of this crucial role of the *WDR48* gene, and the association that we found of a low expression of *WDR48* with poor prognosis, a defective Fanconi Anemia repair pathway may play a role in the malignant transformation of UM. Murine fibroblasts deficient in UAF1 have been shown to exhibit profound chromosomal instability [62].

*XPC* (Xeroderma Pigmentosum, complementation group C) is the third gene located on chromosome 3p. Its low expression was associated with poor survival in our study. The *XPC* gene, located in the region 3p25.1, encodes a protein that helps to form the XPC repair complex and is involved in the early steps of the DNA Nucleotide Excision Repair (NER) pathway. Mutations in *XPC* that impair the production of the XPC protein are related to Xeroderma Pigmentosum (XP), a rare recessive disorder, which makes patients extremely sensitive to ultraviolet light. This results in the frequent development of skin tumors, mainly in areas of the body exposed to the sun. The XPC protein acts a sensor detecting DNA damage [63,64,65,66]. The association of the low expression of *XPC* with poor survival in UM is interesting, since evidence for the association of ultraviolet light exposure and UM development is inconclusive. However, XPC may play a role that is independent of its direct function related to UV-damage, as evidenced by the association of epigenetic silencing of *XPC* with shorter survival in bladder cancer [67]. The XPC repair complex contains the CETN2 protein, which shows a significantly lower expression in metastasizing UMs in the two validation cohorts of our study (Table 4) [68]. Xeroderma Pigmentosum is associated with a higher risk for ocular malignancies [69].

**Figure 7:**
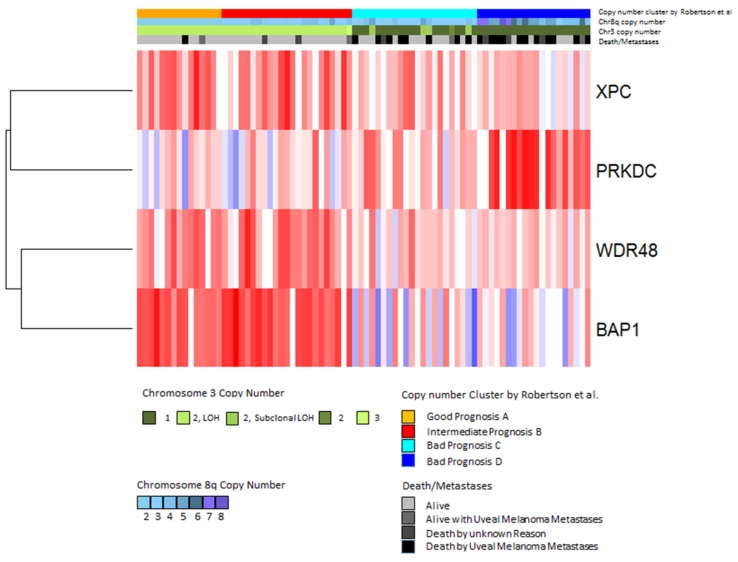
Expression of DNA-repair genes in the TCGA dataset. Gene expression values of the four significantly differentially expressed genes were analyzed by hierarchical clustering using Euclidean distance and average linkage. Each column shows one tumor sample and each row shows one gene. Expression values are shown according to the mean value for each gene (blue = expression below the mean, red = expression above the mean, white = expression at the mean). Subtypes defined by Robertson et al. [42] are indicated in the upper bar above the heatmap as follows: good prognosis (orange), intermediate prognosis (red), bad prognosis group C (cyan) and bad prognosis D (blue) (Jager et al. [75]).

In contrast to the genes discussed above, the *PRKDC* gene that is located on chromosome 8q11.21 was found to be associated with worse survival when highly expressed [70]. A heatmap showing the patients that developed metastases makes it clear that a low BAP1 expression (blue) is associated with a high PRKDC expression (Figure 7). *PRKDC* encodes the catalytic subunit of DNA-dependent serine/threonine protein kinase (DNA-PKcs). DNA-PK is involved in the repair of double-strand breaks (DSBs) by non-homologous end-joining (NHEJ) [71,72,73]. DSBs can develop due to the effects of reactive oxygen intermediates or by exogenous agents such as ionizing radiation and anticancer chemotherapeutic drugs [74].

High expression of DNA-repair proteins such as DNA-PKcs may increase the ability of tumor cells to withstand damage caused by chemotherapy or irradiation. Accordingly, increased DNA-PKcs activity was related to glioma resistance to cisplatin chemotherapy [76]. Moreover, upregulation of DNA-PKcs was detected after irradiation of oral squamous cell carcinoma (OSCC) cells that were resistant to radiotherapy. Targeting DNA-PKcs has been suggested as a novel sensitization therapy of OSCC, and it has been shown to increase anticancer drug sensitivity in osteosarcoma cell lines [77,78]. Since the majority of primary UMs is treated by radiotherapy and certain chemotherapeutic targets are being tested for their effectiveness in killing UM metastases, elucidating the role of DNA-PKcs in UM may pave the way for sensitization therapy in UM by inhibiting DNA-PKcs. While some preliminary results indicate that inhibition of DNA-PKcs by NU7026 sensitizes UM cell lines for the topoisomerase I inhibitor, Topotecan, studies on cervical and breast cancer cells, as well as on lung cancer cells, have shown that this treatment sensitizes tumor cells to radiation treatment [79,80]. As far as we know, this combination has not been tried on UM cells. Van Oorschot et al. showed that the combination of hyperthermia and treatment with NU7441 led to an even better sensitization [79].

We demonstrate that gain of chromosome 8q is related to a higher expression of *PRKDC* in our cases, as well as in the TCGA cohort and UM cell lines. It is known that amplification of chromosome 8q is associated with an adverse clinical outcome in UM [25,81]. Although the exact mechanisms by which gain of chromosome 8q confers its malignant effect has not yet been elucidated, overexpression of *DDEF1* has been suggested as one potential mechanism [82]. A recent study in prostate cancer has shown that the DNA-PKcs protein encoded by *PRKDC* modulates cell invasion and migration and acts as a strong driver of tumor progression and metastasis [45]. In addition, activated DNA-PKcs has been correlated with increased proliferation, decreased apoptosis and poor survival in hepatocellular carcinoma [46]. In accordance, DNA-PKcs has been shown to be involved in normal cell cycle progression by controlling proper chromosome segregation and cytokinesis [83].

In this study, we show that inhibition of DNA-PKcs results in decreased proliferation of UM cells. A recent study by Kotula et al. in the cutaneous melanoma cell line SK28 demonstrated that DNA-PKcs has pro-metastatic activity by modulating the tumor microenvironment through controlling the secretion of, e.g., matrix metalloproteinases (MMPs) and tissue inhibitors of matrix metalloproteinases (TIMPs) [84]. We found a low and variable expression of MMPs and TIMPs in the majority of UM cell lines we analyzed and we did not observe an evident regulatory effect following DNA-PKcs inhibition. Since DNA-PKcs is postulated to be a driver of invasion and metastasis, we analyzed the effect of DNA-PKcs inhibition on the expression of an epithelial-to-mesenchymal transformation (EMT)—associated factors that have been shown to play a role in the invasiveness of UM cells (*ZEB1*, *TWIST1*, *SNAIL1*) [47]. Although the basal expression of these factors was low in the UM cell lines, we observed a decrease in the expression of the pro-metastatic *SNAIL1* upon DNA-PKcs inhibition. The inhibition of the protein interaction between DNA-PKCs and Snail1 has been suggested to be an effective strategy for inhibiting tumor migration [85].

Considering the suggested pro-metastatic functions of DNA-PKcs, it is conceivable that an increased expression of *PRKDC*, as a result of amplification of 8q, may contribute to the malignant progression in UM. This would imply that DNA-PKcs could be a potential target for therapy in UM. Furthermore, the use of inhibitors of DNA-repair proteins is a promising option for treating metastases, since cancer cells only retain some DNA-repair modules and are dependent on these for survival [86].

## 4. Materials and Methods

### 4.1. Study Population

Our ‘training set’ contained 64 UMs obtained by primary enucleation at the Leiden University Medical Center (LUMC), Leiden, The Netherlands, between 1999 and 2008. Patient and tumor characteristics are shown in Table 1. Sufficient frozen material of these tumors was available and DNA of adequate quality could be retrieved. Survival data was retrieved from the patients’ charts and from the Netherlands Comprehensive Cancer Organisation (https://iknl.nl/over-iknl/about-iknl), and updated in March 2017. In The Netherlands, general physicians report every cancer patient to the Netherlands Comprehensive Cancer Organisation, which collects and registers information on the survival status by contacting the general physicians yearly. The follow-up in The Netherlands is not intensive because of a lack of effective treatments for UM metastases and patients are often referred back to their general physician after treatment of the primary tumor. The median follow-up time was 62 months and no patient was lost to follow-up.

Validation of the data was performed using two independent cohorts of post enucleation surgery patients: microarray datasets from Genoa and Paris, and RNAseq data of The Cancer Genome Atlas (TCGA) project [42,75]. Sixty-three untreated uveal melanoma provided by the Biological Resource Centre of Institut Curie (GSE2213840) [86] and 48 UM samples from the Genoa cohort (GSE2783141 and GSE5188042) [41,87] were obtained from the Gene Expression Omnibus (www.ncbi.nlm.nih.gov/geo/). The datasets were combined and normalized as described.

The study followed the tenets of the Declaration of Helsinki (World Medical Association of Declaration 1964; ethical principles for medical research involving human subjects) and the Medical Ethics Committee of the LUMC, Leiden, The Netherlands, had no objection regarding this research (G16.076/NV/gk).

### 4.2. Histologic Examination

After opening the enucleated bulbus, a part of the tumor was retrieved and snap frozen at −80 °C. The remaining tumor tissue was formalin fixed (4% neutral-buffered) and embedded in paraffin. A conventional histologic evaluation by an ophthalmic pathologist for confirmation of diagnosis and determination of characteristics was done. Parameters such as largest basal diameter (LBD, in millimeters), thickness (in millimeters), mitotic count (per 2mm^2^ at 40× magnification, 8 high-power fields), tumor location, cell type (assessed according to the Armed Forces Institute of Pathology atlas) [88] were evaluated on 4 μm-thick hematoxylin and eosin-stained sections. The 8th edition of the AJCC Cancer Staging Manual [89] was used to stage tumors according to the TNM classification system.

### 4.3. Genetic Analyses

DNA and RNA were isolated from fresh-frozen tissue. DNA for single nucleotide polymorphism (SNP) analysis was extracted with the QIAmp DNA Mini kit and RNA for gene-expression profiling with the RNeasy Mini Kit (both from Qiagen, Venlo, The Netherlands). SNP array analysis to determine the chromosome copy number was performed with the Affymetrix 250K_NSP microarray chip (Affymetrix, Santa Clara, CA, USA) on all 64 UMs and with the Affymetrix Cytoscan HD chip (Affymetrix) on the cell lines. The Chromosome Analysis Suite (ChAS, version 2.0225) from Affymetrix was used to determine chromosome copy numbers. Gene-expression profiling at the transcriptional level was carried out on RNA of 64 UMs using 35,244 probes from the Illumina HT-12v4 chip (Illumina, San Diego, CA, USA).

RNA for real-time PCR analysis in cell lines was isolated using the SV total RNA isolation kit (Promega, Madison, WI, USA), then cDNA was synthesized using the reverse transcriptase reaction mixture, as indicated by Promega. qPCR was performed using SYBR green mix (Roche Diagnostics, IN, USA) in a C1000 touch Thermal Cycler (Bio-Rad laboratories, Hercules, CA, USA). Relative expression of PRKDC and SNAIL1 was determined compared to housekeeping genes CAPNS1 and SRPR. The untreated samples average was set at 1.

RNAseq analysis in the cell lines was conducted at Institut Curie (Paris, France) after isolation of total RNA using a NucleoSpin Kit (Macherey-Nagel, Düren, Germany). cDNA synthesis was conducted with MuLV Reverse Transcriptase in accordance with the manufacturers’ instructions (Invitrogen, Carlsbad, CA, USA), with quality assessments conducted on an Agilent (Santa Clara, CA, USA) 2100 Bioanalyzer. Libraries were constructed using the TruSeq Stranded mRNA Sample Preparation Kit (Illumina) and sequenced on an Illumina HiSeq 2500 platform using a 100 bp paired-end sequencing strategy. TopHat (v2.0.6) was used to align the reads against the human reference genome Hg19 RefSeq (RNA sequences, GRCh37) downloaded from the UCSC Genome Browser (http://genome.ucsc.edu). Gene expression was determined by featureCounts and normalized using DESeq2.

Heatmaps and hierarchical clustering were performed in R (heatmap.plus package) using Euclidean distance and average linkage. Gene expression data were also correlated with Uveal Melanoma subtypes according to Robertson et al. [42].

### 4.4. Gene Selection Procedure

We identified 121 genes encoding proteins involved in DNA repair mechanisms, based on a literature review on DNA repair, using the platforms Gene, Online Inheritance in Man (OMIM), Kyoto Encyclopedia of Genes and Genomes (KEGG) and PubMed. As our goal was to identify genes with a variable expression level, we determined the standard deviations of the expression levels of the DNA repair gene probes on the Illumina chip (*n* = 178) (Appendix Table A1). Certain genes were analyzed multiple times because they are encoded by different Illumina probes (in that case, the distinction between probes is made by placing letters in alphabetic order at the end of the gene name), while 18 genes were not analyzed since they were not on the Illumina chip. A selection of genes was made based on a cut-off value of the standard deviation of the expression (Figure 1). A cut-off value of >0.5 would result in 6 genes, of >0.4 in 15 genes, and a cut-off value of >0.3 would lead to a total of 44 genes (encoded by 49 probes). A cut-off value of >0.3 was chosen to have a reasonably-sized group of genes with an acceptable level of variation in expression. The median expression of the probes of these 44 genes was compared between disomy 3 (D3) and monosomy 3 (M3) tumors and corrected for multiple testing using the Bonferroni method. A total of 13 Genes which were significantly differentially expressed after Bonferroni correction were selected for further analysis.

### 4.5. Cell Lines, DNA-PKcs Inhibition, and Proliferation Assay

Cell lines OMM2.5 (originally called OMM1.5 derived from a liver metastasis) and Mel270, which are derived from the same patient, were obtained from Dr. Bruce Ksander [90] and maintained in RPMI supplemented with 10% FBS (fetal bovine serum) and antibiotics. MM28 was obtained from Dr. Sergio Roman-Roman [91] and grown in IMDM supplemented with 20% FBS and antibiotics. The OMM1 cell line, maintained in RPMI supplemented with 10% FBS and antibiotics was established by Dr. Gré Luyten [92]. Cell line 92.1 was developed in Leiden by Dr. Martine Jager [93]. MM28 cells lack BAP1 expression, whereas Mel270, OMM2.5, and OMM1 cells are BAP1-positive.

To evaluate the effect of DNA-PKcs inhibition on the expression of pro-metastatic factors, the expression of these factors was evaluated in a primary UM cell line (Mel270) and in a metastatic UM cell line (MM28) before and after treating the cells with 10 µM NU7026 (#13308, Cayman Chemical, Ann Arbor, MI, USA, stock concentration 20 mM in DMSO) for 5 days. In order to analyze the effect of the DNA-PKcs inhibitor on the growth of these UM cell lines, the cells were seeded in triplicate in 96-well plates. Treatment with NU7026 was started the next day. Cells were replenished with fresh medium with or without drugs after three days. Relative survival was determined after five days with the use of the CellTitre-Blue cell viability assay (Promega) according to the manufacturer’s protocol.

### 4.6. Statistical Analysis

For data analysis, we used the statistical programming language R version 3.0.1 (R: A Language and Environment for Statistical Computing, R Core Team, R foundation for Statistical Computing, Vienna, Austria, 2014, http://www.R-project.org) supplemented with specialized packages for SNP and RNA analysis. The main package used for SNP analysis was aroma.affymetrix, supported by ‘DNAcopy’ (Venkatraman E. Seshan and Adam Olshen, DNAcopy: DNA copy number data analysis. R package version 1.34.0), ‘sfit’ (Henrik Bengtsson and Pratyaksha Wirapati (2013), sfit: Multidimensional simplex fitting. R package version 0.3.0/r185, http://R-Forge.R-project.org/projects/matrixstats/), and ‘R.utils’ (Henrik Bengtsson (2014), R.utils: Various programming utilities, R package version 1.29.8, http://CRAN.R-project.org/package=R.utils). The ‘Aroma.Affymetrix’ package made it possible to use the information from the SNP microarrays to determine copy number values [94,95].

The packages used for RNA microarray analysis were ‘limma’ version 3.16.8, and the specific packages for Illumina microarrays: ‘lumi’ version 2.12.0, ‘annotate’ (R. Gentleman, annotate: Annotation for microarrays, R package version 1.38.0), and the database package ‘IlluminaHumanv4.db’ (Mark Dunning, Andy Lynch and Matthew Eldridge, IlluminaHumanv4.db: Illumina HumanHT12v4 annotation data (chip IlluminaHumanv4), R package version 1.18.0).

The statistical software package SPSS v.20.0.0 (IBM SPSS Statistics for Windows, IBM Corp., Armonk, NY, USA) was used for data analysis. Population characteristics were described using medians and percentages. The Mann–Whitney U test was performed to analyze numerical variables between two groups, and the Kruskal–Wallis test in case more than two groups were compared. Kaplan–Meier survival curves were made and the log rank test was used to analyze significance. Differences were considered to be significant if *p* < 0.05 after correction for multiple testing.

## 5. Conclusions

We show that several important DNA-repair molecules are differentially expressed between tumors with good and adverse prognosis. Furthermore, we report on the effects of DNA-PKcs inhibition on cell survival and expression of pro-metastatic genes in UM cell lines. We suggest that DNA-PKcs, encoded by the *PRKDC* gene on chromosome 8q, may be involved in proliferation, invasion, and metastasis of UM cells and should be investigated further. An increased insight of factors involved in DNA repair mechanisms in uveal melanoma will hopefully enhance our understanding of the pathogenesis of this disease and may eventually result in the identification of new targets of therapy.

## Figures and Tables

**Table 1:** Baseline characteristics of the test and validation sets. Percentages are rounded and may not equal 100.

CHARACTERISTIC	LUMC COHORT (*n* = 64) Test Set	GENOA and PARIS COHORT (*n* = 110) Validation Set	TCGA COHORT (*n* = 80) 2nd Validation Set
**Gender**			
Female	31 (48%)	41 (38%)	35 (44%)
Male	33 (52%)	67 (62%)	45 (56%)
**Median age at enucleation/diagnosis (TCGA)** (range)	61.6 (12.8–88.4) years	63.0 (29.0–85.0) years	61.5 (22.0–86.0) years
**Median LBD** (range)	13.0 (8.0–30.0) mm	15.0 (2.0–23.0) mm	16.8 (10.0–23.6) mm
**Median prominence** (range)	8.0 (2.0–12.0) mm	11.1 (3.0–17.0) mm	11.0 (4.4–16.0) mm
**AJCC size categories**			
T1	6 (9%)	1 (1%)	0 (0%)
T2	25 (39%)	24 (27%)	14 (18%)
T3	31 (48%)	39 (44%)	32 (40%)
T4	2 (3%)	25 (28%)	34 (43%)
**Cell type**			
Spindle	22 (34%)	10 (12%)	43 (54%)
Mixed/epithelioid	42 (66%)	71 (88%)	37 (46%)
**Chromosome 3 status**			
No monosomy 3	24 (38%)	46 (48%)	43 (54%) *
Monosomy 3	40 (63%)	49 (52%)	37 (46%)
**Metastasis**			
No	27 (42%)	54 (49%)	53 (66%)
Yes	37 (58%)	56 (51%)	27 (34%)

* Four tumors were isodisomy 3. Abbreviations: AJCC: American Joint Committee on Cancer; LBD: largest basal diameter; mm: millimeters; *n*: number of patients.

**Table 2:** Differentially-expressed genes in relation to chromosome 3 status. Only significantly differentially expressed genes between monosomic and disomic chromosome 3 UMs are shown. Table 2A shows the genes that had a higher expression in tumors with monosomy 3, and Table 2B shows the tumors with a lower expression in tumors with monosomy 3. The Mann–Whitney U test and Bonferroni correction were applied. BER: base excision repair; DSBR: double-strand break repair; FA: Fanconi Anemia; MMR: mismatch repair; NER: nucleotide excision repair.

A. Higher expression in Monosomy 3 tumors						
GENE	**CHARACTERISTICS OF GENE**		**EXPRESSION Median (Range)**		***p*-VALUES**	
	**Pathway**	**Chromosome Location**	**Disomy 3 (*n* = 24)**	**Monosomy 3 (*n* = 40)**	***p*-Value**	**Corrected *p*-Value**
**CENPX**	FA	17q25.3	9.3 (8.9–10.3)	9.7 (9.0–10.6)	<0.001	<0.001
**DDB1**	NER	11q12.2	12.1 (11.3–13.0)	12.4 (11.7–13.0)	0.001	0.04
**PRKDC**	DSBR	8q11.21	8.0 (7.3–8.6)	8.8 (7.8–10.2)	<0.001	<0.001
B. Lower expression in Monosomy 3 tumors						
GENE	**CHARACTERISTICS OF GENE**		**EXPRESSION Median (Range)**		***p*-VALUES**	
	**Pathway**	**Chromosome Location**	**Disomy 3 (*n* = 24)**	**Monosomy 3 (*n* = 40)**	***p*-Value**	**Corrected *p*-Value**
**APEX1**	BER	14q11.2	11.0 (9.9–11.4)	10.5 (9.6–11.4)	<0.001	0.004
**BAP1**	DSBR	3p21.1	8.0 (6.6–8.5)	7.4 (6.4–8.1)	<0.001	<0.001
**CETN2**	NER	Xq28	10.2 (9.7–11.2)	9.9 (9.3–10.7)	<0.001	0.002
**GTF2H4**	NER	6p21.33	8.5 (6.9–9.4)	7.9 (7.2–9.3)	<0.001	<0.001
**MLH1**	MMR/FA	3p22.2	8.2 (7.5–8.8)	7.8 (7.1–8.3)	<0.001	<0.001
**RMI2**	DSBR	16p13.13	7.2 (6.7–7.7)	6.9 (6.5–7.7)	<0.001	0.02
**RPA1**	DSBR/MMR/NER	17p13.3	8.7 (7.7–9.2)	8.3 (7.4–8.9)	0.001	0.04
**SEM1**	DSBR	7q21.3	7.7 (7.3–8.4)	7.4 (6.8–8.0)	<0.001	0.01
**WDR48**	FA	3p22.2	8.2 (7.4–8.6)	7.6 (7.2–8.2)	<0.001	<0.001
**XPC**	NER	3p25.1	9.2 (8.3–9.7)	8.6 (8.0–9.3)	<0.001	<0.001

**Table 3:** Relationship between chromosome dose and gene expression for all 44 genes of interest. The analysis was reliable only for genes located on chromosomes 3, 6 or 8, since SNP analyses of other chromosomes showed no aberrant copy number in most tumors.

GENE	CHARACTERISTICS OF GENE		EXPRESSION Median (Range)		*p*-Value
	Pathway	Chromosome Location	No Aberrant Copy Number	Aberrant Copy Number	
**BAP1**	DSBR	3p21.1	8.0 (6.6–8.5) *n = 24*	7.4 (6.4–8.1) *n = 40*	<0.001
**FANCE**	FA/DSBR	6p21.31	7.4 (6.8–8.2) *n = 43*	7.9 (6.9–8.6) *n = 21*	<0.001
**GTF2H4**	NER	6p21.33	7.9 (6.9–8.5) *n = 43*	8.5 (7.8–9.4) *n = 21*	<0.001
**GTF2H5**	NER	6q25.3	10.3 (9.4–11.2) *n = 53*	9.9 (9.3–10.4) *n = 11*	0.004
**MBD4**	BER/DSBR	3q21.3	8.4 (7.5–9.9) *n = 24*	8.1 (7.5–9.3) *n = 40*	0.33
**MLH1**	MMR/FA	3p22.2	8.2 (7.5–8.8) *n = 24*	7.8 (7.1–8.3) *n = 40*	<0.001
**NBN**	DSBR	8q21.3	7.9 (7.3–8.3) *n = 19*	8.2 (7.4–9.2) *n = 45*	<0.001
**POLB**	BER	8p11.21	10.0 (8.6–10.9) *n = 49*	8.9 (8.1–10.2) *n = 15*	<0.001
**PRKDC**	DSBR	8q11.21	8.0 (7.5–8.5) *n = 19*	8.7 (7.3–10.2) *n = 45*	<0.001
**WDR48**	FA	3p22.2	8.2 (7.4–8.6) *n = 24*	7.6 (7.2–8.2) *n = 40*	<0.001
**XPC**	NER	3p25.1	9.2 (8.3–9.7) *n = 24*	8.6 (8.0–9.3) *n = 40*	<0.001

Chromosome 3: loss; chromosome 6p: gain; chromosome 6q; loss; chromosome 8p: loss; chromosome 8q: gain. Abbreviations: BER: base excision repair; DSBR: double-strand break repair; FA: Fanconi anemia; MMR: mismatch repair; NER: nucleotide excision repair.

**Table 4:** Validation of the 13 significantly differentially expressed genes between disomy 3 and monosomy 3 tumors in the LUMC cohort. Validation was performed in an independent cohort of 110 tumors (Genoa + Paris) and in the TCGA cohort of 80 tumors. *p*-values of the log-rank test are shown. Significant *p*-values are in bold. Genes that are significantly associated with survival in all cohorts are depicted in the last column. Abbreviations: BER: base excision repair; DSBR: double-strand break repair; FA: Fanconi Anemia; MMR: mismatch repair; NER: nucleotide excision repair.

GENE	CHARACTERISTICS OF GENE		LUMC COHORT (*n* = 64) Test Set	GENOA & PARIS COHORT (*n* = 110) Validation Set	TCGA COHORT (*n* = 80) 2nd Validation Set	Validated Genes
	Pathway	Chromosome Location				
**CENPX**	FA	17q25.3	**<0.001**	0.09	**0.03**	
**DDB1**	NER	11q12.2	0.48	0.75	0.22	
**PRKDC**	DSBR	8q11.21	**0.001**	**0.005/0.01/<0.001**	**0.002**	***PRKDC*** *
**APEX1**	BER	14q11.2	0.05	0.77	**0.04**	
**BAP1**	DSBR	3p21.1	**0.001**	**<0.001/**0.15	**<0.001**	***BAP1*** †
**CETN2**	NER	Xq28	0.18	**0.04**	**0.001**	
**GTF2H4**	NER	6p21.33	**0.001**	0.97	**0.001**	
**MLH1**	MMR/FA	3p22.2	0.07	**0.005**	0.08	
**RMI2**	DSBR	16p13.13	**0.02**	0.63	**0.005**	
**RPA1**	DSBR/MMR/NER	17p13.3	0.41	0.39/0.26/**0.002**	**0.04**	
**SEM1**	DSBR	7q21.3	**0.006**	0.06	**0.02**	
**WDR48**	FA	3p22.2	**<0.001**	0.07/0.06/**0.04/0.03**	**0.003**	***WDR48*** †
**XPC**	NER	3p25.1	**0.005**	**0.02**	**0.01**	***XPC*** †

Symbols: * = higher expression is associated with poor survival. † = lower expression is associated with poor survival.

## Appendix A

**Table A1:** Alphabetic list of all DNA repair genes (*n* = 121, encoded by 178 probes) evaluated in our cohort. The expression of genes with a standard deviation >0.3 (*n* = 44, encoded by 49 probes) was compared between disomy 3 (*n* = 24) and monosomy (*n* = 40) tumors. Bonferroni correction was applied to the unrounded p-values. Significant corrected *p*-values and corresponding probes are in bold.

GENE	MEAN EXPRESSION	STANDARD DEVIATION	DISOMY 3 (*n* = 24) Median (Range)	MONOSOMY 3 (*n* = 40) Median (Range)	*p*-VALUE	CORRECTED *p*-VALUE
**APEX1**	9.76	0.58	10.2 (8.3–10.8)	9.6 (8.5–10.7)	0.001	0.07
**APEX1a**	10.69	0.42	11.0 (9.9–11.4)	10.5 (9.6–11.4)	<0.001	**0.004**
**APEX2**	7.87	0.21				
**APITD1**	Not in Illumina	Not in Illumina				
**ATR**	Not in Illumina	Not in Illumina				
**ATRIP**	7.33	0.22				
**ATRIPa**	7.12	0.21				
**BAP1**	7.52	0.53	8.0 (6.6–8.5)	7.4 (6.4–8.1)	<0.001	**<0.001**
**BIVM-ERCC5**	7.46	0.28				
**BIVM-ERCC5a**	7.82	0.31	7.9 (7.4–8.3)	7.8 (7.1–8.5)	0.05	1
**BLM**	6.46	0.11				
**BRCA1**	6.49	0.15				
**BRCA1a**	6.73	0.16				
**BRCA2**	Not in Illumina	Not in Illumina				
**BRIP1**	6.47	0.1				
**C17orf70**	9.1	0.39	9.0 (8.3–9.8)	9.2 (8.3–10.4)	0.02	0.84
**C19orf40**	7.06	0.19				
**CCNH**	7.54	0.34	7.4 (6.8–8.2)	7.6 (7.0–8.4)	0.008	0.38
**CCNHa**	7.66	0.22				
**CDK7**	8.89	0.39	8.8 (7.7–9.7)	8.9 (8.4–10.3)	0.21	1
**CENPX**	9.61	0.38	9.3 (8.9–10.3)	9.7 (9.0–10.6)	<0.001	**<0.001**
**CETN2**	10.02	0.38	10.2 (9.7–11.2)	9.9 (9.3–10.7)	<0.001	**0.002**
**CUL4B**	7.71	0.27				
**CUL4Ba**	6.53	0.17				
**DCLRE1C**	6.7	0.14				
**DCLRE1Ca**	7.28	0.32	7.2 (6.7–8.4)	7.3 (6.9–8.3)	0.31	1
**DDB1**	12.33	0.37	12.1 (11.3–13.0)	12.4 (11.7–13.0)	0.001	**0.04**
**DDB1a**	6.63	0.18				
**DDB1b**	6.9	0.18				
**DDB1c**	8.27	0.28				
**DDB2**	6.95	0.23				
**DNTT**	Not in Illumina	Not in Illumina				
**EME1**	6.54	0.1				
**EME2**	Not in Illumina	Not in Illumina				
**ERCC1**	9.52	0.27				
**ERCC1a**	7.38	0.3				
**ERCC1b**	9.74	0.29				
**ERCC1c**	6.53	0.11				
**ERCC2**	7.79	0.28				
**ERCC3**	7.98	0.17				
**ERCC3a**	6.6	0.11				
**ERCC4**	6.47	0.09				
**ERCC6**	6.48	0.11				
**ERCC8**	6.62	0.13				
**ERCC8a**	6.52	0.12				
**EXO1**	6.62	0.25				
**EXO1a**	6.39	0.13				
**FAN1**	8.71	0.28				
**FAN1a**	7.1	0.16				
**FANCA**	6.5	0.12				
**FANCAa**	6.52	0.11				
**FANCAb**	6.36	0.13				
**FANCB**	6.4	0.12				
**FANCC**	Not in Illumina	Not in Illumina				
**FANCD2**	6.74	0.2				
**FANCE**	7.59	0.36	7.7 (6.8–8.6)	7.4 (7.1–8.3)	0.003	0.13
**FANCF**	Not in Illumina	Not in Illumina				
**FANCG**	7.48	0.34	7.4 (7.0–8.5)	7.4 (6.9–8.4)	0.69	1
**FANCI**	6.59	0.18				
**FANCL**	6.42	0.14				
**FANCLa**	7.78	0.4	7.9 (7.1–8.6)	7.6 (6.9–8.9)	0.06	1
**FANCM**	Not in Illumina	Not in Illumina				
**FEN1**	7.09	0.19				
**FEN1a**	8.97	0.32	9.0 (8.4–9.7)	9.0 (8.1–9.6)	0.98	1
**GTF2H1**	6.91	0.19				
**GTF2H1a**	7.42	0.25				
**GTF2H2**	6.27	0.13				
**GTF2H2B**	7.13	0.32	7.0 (6.6–7.9)	7.2 (6.5–8.1)	0.37	1
**GTF2H3**	7.12	0.19				
**GTF2H4**	8.09	0.47	8.5 (6.9–9.4)	7.9 (7.2–9.3)	<0.001	**<0.001**
**GTF2H5**	10.19	0.43	10.1 (9.3–11.0)	10.3 (9.3–11.2)	0.01	0.62
**LIG1**	8.27	0.31	8.2 (7.6–8.7)	8.3 (7.8–9.0)	0.09	1
**LIG3**	6.85	0.12				
**LIG3a**	7.18	0.25				
**LIG4**	6.63	0.08				
**LIG4a**	6.47	0.11				
**MBD4**	8.26	0.46	8.4 (7.5–9.9)	8.1 (7.5–9.3)	0.33	1
**MGMT**	9.34	0.43	9.6 (8.7–10.3)	9.2 (8.3–10.1)		1
**MLH1**	7.94	0.33	8.2 (7.5–8.8)	7.8 (7.1–8.3)	<0.001	**<0.001**
**MLH3**	6.44	0.11				
**MLH3a**	6.63	0.15				
**MNAT1**	6.8	0.2				
**MPG**	6.61	0.09				
**MPGa**	6.31	0.12				
**MRE11A**	6.59	0.08				
**MRE11Aa**	6.74	0.17				
**MSH2**	6.46	0.12				
**MSH2a**	6.68	0.12				
**MSH3**	7.72	0.26				
**MSH3a**	13.39	0.65	13.3 (12.1–14.7)	13.5 (12.3–15.0)	0.13	1
**MSH6**	8.8	0.3				
**MUS81**	7.76	0.22				
**MUTYH**	6.61	0.12				
**MUTYHa**	7.85	0.38	7.9 (7.3–8.4)	7.9 (6.9–8.5)	0.39	1
**MUTYHb**	6.78	0.19				
**NBN**	8.1	0.42	7.9 (7.4–8.5)	8.2 (7.3–9.2)	0.005	0.24
**NBNa**	7.01	0.25				
**NEIL1**	6.58	0.11				
**NEIL2**	8.06	0.45	8.3 (7.4–8.8)	7.9 (6.9–9.1)	0.002	0.1
**NHEJ1**	Not in Illumina	Not in Illumina				
**NEIL3**	Not in Illumina	Not in Illumina				
**NTHL1**	7.76	0.31	7.8 (7.1–8.5)	7.7 (7.2–8.3)	0.01	0.64
**OGG1**	6.48	0.14				
**OGG1a**	6.94	0.2				
**PALB2**	7.21	0.19				
**PARP2**	7.43	0.22				
**PARP2a**	6.89	0.16				
**PCNA**	6.58	0.16				
**PCNAa**	8.53	0.51	8.3 (7.7–9.7)	8.6 (7.5–9.9)	0.002	0.12
**PMS2**	6.97	0.21				
**PMS2a**	6.44	0.15				
**PMS2CL**	6.79	0.14				
**PMS2CLa**	6.48	0.11				
**POLB**	9.72	0.63	9.9 (9.1–10.7)	9.7 (8.1–10.9)	0.06	1
**POLD3**	6.77	0.13				
**POLE3**	9.62	0.31	9.7 (8.8–10.3)	9.6 (9.1–10.3)	0.24	1
**POLH**	6.52	0.13				
**POLHa**	6.55	0.09				
**POLI**	Not in Illumina	Not in Illumina				
**POLK**	Not in Illumina	Not in Illumina				
**POLL**	6.89	0.2				
**POLM**	7.14	0.22				
**POLN**	6.53	0.22				
**PRKDC**	6.64	0.21				
**PRKDCa**	8.55	0.68	8.0 (7.3–8.6)	8.8 (7.8–10.2)	<0.001	**<0.001**
**PRKDCb**	6.64	0.15				
**PRKDCc**	6.54	0.12				
**PRKDCd**	6.56	0.1				
**RAD23A**	9.77	0.24				
**RAD50**	7.59	0.19				
**RAD51**	6.97	0.26				
**RAD51a**	6.81	0.13				
**RAD51C**	Not in Illumina	Not in Illumina				
**RAD52**	Not in Illumina	Not in Illumina				
**RAD54B**	6.52	0.1				
**RAD54Ba**	6.81	0.16				
**RBX1**	10.17	0.39	10.0 (9.4–10.8)	10.2 (9.6–11.1)	0.01	0.55
**REV1**	7.78	0.17				
**REV1a**	7.75	0.19				
**REV3L**	6.66	0.2				
**RFC1**	7.37	0.19				
**RFC1a**	8.66	0.28				
**RMI1**	6.84	0.14				
**RMI1a**	7.27	0.2				
**RMI2**	7.07	0.32	7.2 (6.7–7.7)	6.9 (6.5–7.7)	<0.001	**0.02**
**RPA1**	8.2	0.34	8.5 (7.7–8.9)	8.1 (7.4–8.9)	0.006	0.28
**RPA1a**	9.74	0.36	9.9 (8.7–10.4)	9.7 (8.9–10.5)	0.02	1
**RPA1b**	8.39	0.36	8.7 (7.7–9.2)	8.3 (7.4–8.9)	0.001	**0.04**
**RPA2**	9.95	0.38	10.1 (9.3–10.9)	10.0 (9.2–10.7)	0.22	1
**RPA3**	9	0.41	9.1 (8.5–10.1)	8.9 (8.0–9.9)	0.09	1
**RPA4**	6.38	0.13				
**SEM1**	11.32	0.24				
**SEM1a**	7.53	0.32	7.7 (7.3–8.4)	7.4 (6.8–8.0)	<0.001	**0.01**
**SLX1A**	Not in Illumina	Not in Illumina				
**SLX1B**	Not in Illumina	Not in Illumina				
**SLX4**	6.96	0.19				
**SMUG1**	9.04	0.23				
**TELO2**	6.96	0.29				
**TDG**	Not in Illumina	Not in Illumina				
**TOP3A**	7.39	0.36	7.3 (6.7–8.6)	7.4 (6.9–8.3)	0.22	1
**TOP3Aa**	6.78	0.15				
**TOP3B**	7.89	0.26				
**UBE2T**	7.32	0.37	7.3 (6.8–8.2)	7.3 (6.7–8.5)	0.49	1
**UNG**	6.31	0.12				
**UNGa**	9.6	0.41	9.6 (8.8–10.5)	9.7 (8.7–10.3)	0.53	1
**UNGb**	6.54	0.11				
**USP1**	7.57	0.29				
**USP1a**	6.48	0.12				
**WDR48**	7.82	0.36	8.2 (7.4–8.6)	7.6 (7.2–8.2)	<0.001	**<0.001**
**XPA**	6.99	0.14				
**XPC**	8.76	0.42	9.2 (8.3–9.7)	8.6 (8.0–9.3)	<0.001	**<0.001**
**XRCC1**	7.88	0.3	7.9 (7.3–8.7)	7.9 (7.3–8.5)	0.57	1
**XRCC4**	Not in Illumina	Not in Illumina				
**XRCC5**	8.94	0.32	9.0 (8.2–9.6)	9.0 (8.1–9.6)	0.95	1
**XRCC6**	8.5	0.31	8.3 (7.8–8.8)	8.6 (7.7–9.3)	0.005	0.26
**XRCC6a**	6.73	0.13				
**XRCC6b**	8.75	0.38	8.7 (7.9–9.2)	8.8 (7.9–10.0)	0.65	1
**XRCC6c**	10.61	0.37	10.5 (9.5–11.2)	10.7 (9.8–11.2)	0.02	0.87

## Appendix B

**Table A2:** Association of the 13 significantly differentially expressed genes between disomy 3 and monosomy 3 tumors, with clinicopathologic parameters and survival. Significant *p*-values are indicated in bold.

A: Highly expressed genes in monosomy 3 tumors.										
**CHARACTERISTIC**	**GENE**									
	CENPX (17q25.3)			DDB1 (11q12)			PRKDC (8q11.21)			
	**PATHWAY**									
	FA			NER			DSBR			
**Largest Basal Diameter**										
≤13 mm (*n* = 34) *median(range)*	9.4 (9.0–10.6)			12.3 (11.3–13.0)			8.4 (7.5–9.8)			
>13 mm (*n* = 30) *median(range)*	9.7 (8.9–10.5)			12.3 (11.7–12.8)			8.7 (7.3–10.2)			
*p*-value *	**0.02**			0.93			0.11			
**Cell type**										
Spindle (*n* = 22) *median (range)*	9.5 (8.9–10.4)			12.3 (11.7–13.0)			8.2 (7.3–9.7)			
Mixed/epithelioid (*n* = 42) *median(range)*	9.6 (9.0–10.6)			12.3 (11.3–13.0)			8.6 (7.5–10.2)			
*p*-value *	**0.04**			0.59			0.12			
**AJCC Stage**										
Stage I (*n* = 5) *median (range)*	9.3 (9.0–9.9)			12.6 (12.2–13.0)			8.2 (7.7–9.6)			
Stage II (*n* = 36) *median (range)*	9.5 (8.9–10.6)			12.3 (11.3–13.0)			8.4 (7.5–9.8)			
Stage III (*n* = 23) *median (range)*	9.7 (9.3–10.5)			12.3 (11.8–12.9)			8.7 (7.3–10.2)			
*p*-value †	**0.01**			0.1			0.19			
**Presence of metastases**										
No (*n* = 27) *median (range)*	9.3 (9.0–10.4)			12.3 (11.2–13.0)			8.1 (7.5–9.4)			
Yes (*n* = 37) *median (range)*	9.8 (8.9–10.6)			12.4 (11.7–13.0)			8.8 (7.3–10.2)			
*p*-value *	**<0.001**			0.3			**<0.001**			
**Survival analysis**										
Expression lower than or equal to median	≤9.6			≤12.3			≤8.4			
Expression higher than median	>9.6 §			>12.3			>8.4 §			
*p*-value ‡	**<0.001**			0.48			**0.001**			
B: Lowly expressed genes in monosomy 3 tumors.										
CHARACTERISTIC	**GENE**									
	APEX1 (14q11.2)	BAP1 (3p21.1)	CETN2 (Xq28)	GTF2H4 (6p21.33)	MLH1 (3p22.2)	RMI2 (16p13.13)	RPA1 (17p13.3)	SEM1 (7q21.3)	WDR48 (3p22.2)	XPC (3p25.1)
	**PATHWAY**									
	BER	DSBR	NER	NER	MMR/FA	DSBR	DSBR/MMR/NER	DSBR	FA	NER
**LBD**										
**≤13 mm (*n* = 34) *median(range)***	10.7 (9.9–11.4)	7.7 (6.6–8.5)	10.0 (9.3–11.2)	8.0(6.9–9.1)	8.0(7.5–8.8)	7.09 (6.6–7.7)	8.4 (7.7–9.2)	7.5(7.0–8.4)	8.0(7.2–8.5)	8.9 (8.0–9.6)
**>13 mm (*n* = 30) *median(range)***	10.7 (9.6–11.4)	7.4 (6.4–8.5)	9.9 (9.3–10.9)	8.0(7.2–9.4)	7.9 (7.1–8.6)	7.0 (6.5–7.7)	8.4 (7.4–8.8)	7.4 (6.8–8.0)	7.7 (7.2–8.6)	8.6 (8.0–9.7)
***p*-value ***	0.75	0.17	0.24	0.86	0.16	0.11	0.2	0.11	**0.01**	**0.004**
**Cell type**										
**Spindle (*n* = 22) *median (range)***	10.8 (9.6–11.4)	7.7 (6.6–8.5)	10.1 (9.6–10.9)	8.2 (7.2–9.4)	7.9 (7.1–8.8)	7.1 (6.5–7.7)	8.6 (7.4–9.2)	7.5 (6.8–8.0)	8.0(7.3–8.6)	8.9 (8.0–9.7)
**Mixed/epithelioid (*n* = 42) *median(range)***	10.6 (9.6–11.4)	7.6 (6.4–8.5)	10.0 (9.3–11.2)	8.0 (6.9–9.1)	7.9 (7.2–8.8)	6.9 (6.6–7.7)	8.4(7.5–9.1)	7.5 (6.9–8.4)	7.7 (7.2–8.5)	8.7 (8.0–9.3)
***p*-value ***	0.13	0.15	0.1	0.12	0.97	0.15	0.06	0.09	**0.007**	**0.03**
**AJCC Stage**										
Stage I (*n* = 5) *median (range)*	10.7 (10.4–11.0)	7.7 (7.5–8.1)	10.1 (9.3–11.2)	8.1 (7.9–8.5)	7.8 (7.6–8.4)	7.1 (6.9–7.6)	8.7 (7.9–9.1)	7.6 (7.1–8.2)	7.9 (7.5–8.5)	8.8 (8.6–9.2)
**Stage II (*n* = 36) *median (range)***	10.8 (9.7–11.4)	7.7 (6.4–8.5)	10.0(9.6–10.7)	8.0 (6.9–9.4)	8.0 (7.5–8.8)	7.0 (6.6–7.7)	8.5 (7.7–9.2)	7.5 (7.0–8.4)	8.1 (7.3–8.6)	8.8 (8.0–9.7)
**Stage III (*n* = 23)*median (range)***	10.5 (9.6–11.3)	7.4 (6.6–8.5)	9.9 (9.3–10.9)	8.0 (7.5–9.1)	7.8 (7.1–8.6)	7.1 (6.5–7.7)	8.3 (7.4–8.8)	7.4 (6.8–8.0)	7.7 (7.2–8.3)	8.6 (8.0–9.3)
***p*-value †**	0.1	0.35	0.16	0.76	0.05	0.44	**0.03**	0.27	0.08	0.14
**Presence of metastases**										
**No (*n* = 27) *median (range)***	10.8 (9.9–11.4)	7.8 (6.6–8.5)	10.1 (9.7–11.2)	8.3 (6.9–9.3)	8.1 (7.5–8.8)	7.1(6.7–7.7)	8.4(7.7–9.2)	7.6 (7.3–8.4)	8.1(7.2–8.6)	9.0 (8.0–9.6)
**Yes (*n* = 37) *median (range)***	10.6 (9.6–11.4)	7.4(6.4–8.3)	9.9 (9.3–10.9)	7.9 (7.2–9.4)	7.9 (7.1–8.6)	6.9 (6.5–7.7)	8.4 (7.4–8.9)	7.4 (6.8–8.01)	7.7 (7.2–8.3)	8.6 (8.0–9.7)
***p*-value ***	0.11	**0.003**	**0.03**	**0.005**	**0.02**	**0.003**	**0.09**	**0.006**	**<0.001**	**0.006**
**Survival analysis**										
**Expression ≤ median**	≤10.7	≤7.6 §	≤10	≤8 §	≤7.9	≤7.1 §	≤8.4	≤7.5 §	≤7.8 §	≤8.7 §
**Expression higher than median**	>10.7	>7.6	>10	>8	>7.9	>7.1	>8.4	>7.5	>7.8	>8.7
***p*-value ‡**	0.05	**0.001**	0.18	**0.001**	0.07	**0.02**	0.41	**0.006**	**<0.001**	**0.005**

Abbreviations: BER: base excision repair; DSBR: double-strand break repair; FA: fanconi anemia; MMR: mismatch repair; NER: nucleotide excision repair. Symbols: *: Mann–Whitney U test, †: Kruskal–Wallis test, ‡: Log-rank test, §: worse survival.
